# Case Report: Neuropathic pain in a patient with congenital insensitivity to pain

**DOI:** 10.12688/f1000research.2642.2

**Published:** 2015-06-19

**Authors:** Daniel W. Wheeler, Michael C.H. Lee, E. Katherine Harrison, David K. Menon, C. Geoffrey Woods

**Affiliations:** 1 Division of Anaesthesia, University of Cambridge, Cambridge, CB2 0QQ, UK; 2 School of Clinical Medicine, University of Cambridge, Cambridge, CB2 0SP, UK; 3 Department of Medical Genetics, University of Cambridge, Cambridge, CB2 0QQ, UK

**Keywords:** Pain, Neuropathic Pain, Congenital Insensitivity to Pain, Channelopathy

## Abstract

We report a unique case of a woman with Channelopathy-associated Insensitivity to Pain (CIP) Syndrome, who developed features of neuropathic pain after sustaining pelvic fractures and an epidural hematoma that impinged on the right fifth lumbar (L5) nerve root. Her pelvic injuries were sustained during painless labor, which culminated in a Cesarean section. She had been diagnosed with CIP as child, which was later confirmed when she was found to have null mutations of the *SCN9A* gene that encodes the voltage-gated sodium channel Nav1.7. She now complains of troubling continuous buzzing in both legs and a vice-like squeezing in the pelvis on walking. Quantitative sensory testing showed that sensory thresholds to mechanical stimulation of the dorsum of both feet had increased more than 10-fold on both sides compared with tests performed before her pregnancy. These findings fulfill the diagnostic criteria for neuropathic pain. Notably, she mostly only experiences the negative symptoms (such as numbness and tingling, but also electric shocks), and she has not reported sharp or burning sensations, although the value of verbal descriptors is somewhat limited in a person who has never felt pain before. However, her case strongly suggests that at least some of the symptoms of neuropathic pain can persist despite the absence of the Nav1.7 channel. Pain is a subjective experience and this case sheds light on the transmission of neuropathic pain in humans that cannot be learned from knockout mice.

## Case

There has been an explosion of interest in Nav1.7 as a potential therapeutic target for novel analgesics, as mutations in *SCN9A* are associated with profoundly altered pain thresholds ^1^. Perhaps the greatest level of interest has been reserved for those very rare individuals with autosomal recessive mutations that truncate the protein Nav1.7 resulting in a complete lack of expression of the ion channel. The result is Channelopathy-associated Insensitivity to Pain (CIP) Syndrome: a complete absence of pain sensation, while all other sensory modalities apart from the sense of smell remain intact. Here we describe the experiences of a Caucasian 37-year-old patient with CIP whose older sister, but neither of her parents or other family members, is also affected. Other than a variety of injuries to the cornea and tongue, burns and relatively minor fractures sustained during childhood and now ascribed to CIP, there was no other medical history of note. Nonetheless, after childbirth she developed symptoms that she now readily describes as pain, and which has neuropathic features. We believe that this case report provides insights into the mechanisms of neuropathic pain, dissecting “positive” from “negative” symptomatology, and shows that it is possible to experience neuropathic pain in the absence of prior experience of acute pain.

Our patient had been recognized as having CIP aged 7, diagnosed at the same time as her older sister and confirmed 18 years later by finding bi-allelic heterozygous null mutations of *SCN9A* in exon 29 (c.4975T>A p.K1659X) and exon 22 (c.3699-3709delATGGATAGCAT p.I1235LfsX2). The *SCN9A* gene on chromosome 2q24.3 encodes the alpha-subunit of the Nav1.7 voltage-gated sodium channel, which is expressed at high levels in small-diameter peripheral nociceptive neurons ^2^.

**Figure 1.**
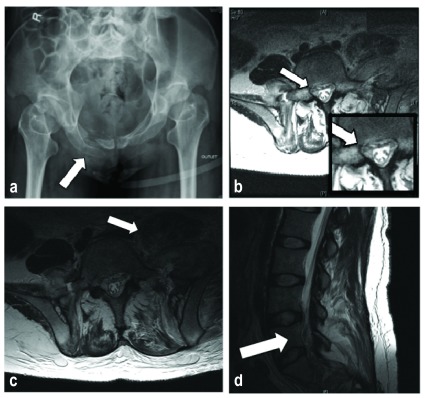
**a**) an anteroposterior X-ray outlet view of the patient’s pelvis showing multiple fractures of the superior and inferior pubic rami; **b**) axial T2-weighted non-enhanced magnetic resonance image plus magnification showing hematoma adjacent to the right L5 nerve root at the exit foramen (arrow); **c**) a more cranial axial view showing the extent of the pelvic hematoma, and **d**) a sagittal view at the right exit foramina showing hematoma around the distal cauda equina.

She sustained painless pelvic fractures, presumably during labor, which were not recognized for two months. By then, examination revealed significant weakness in both legs, worse on the right, and with both ankle reflexes absent. We subsequently compared the results of formal quantitative sensory testing three months post-injury to those obtained four years pre-injury. Sensory thresholds to heat and cold in the foot dorsum were broadly similar on both sides ( Table 1), and should be interpreted in the context of someone who has never felt pain. However, thresholds to mechanical (von Frey) stimulation of the dorsum of both feet were increased more than 10-fold bilaterally. Imaging studies revealed multiple fractures of both sacral wings and of the superior and inferior pubic rami bilaterally ( Figure 1a). Furthermore, there was an extensive hematoma extending into the left iliopsoas, right obturator externus and spinal canal, causing occlusion of the thecal sac at the level of the fifth lumbar (L5) and first sacral (S1) intervertebral space ( Figure 1b, 1c and 1d). The fractures were attributed to transient osteoporosis of pregnancy, and their severity to her continued walking in the face of CIP. However, shortly after the fractures were diagnosed, bone densitometry studies and all serum bone profile results were found to be normal.

**Table 1.** Temperature and mechanical thresholds of the dorsum of both feet before and after childbirth (baseline temperature 32°C).

Body part tested	Dorsum right foot		Dorsum left foot	
Mean threshold	Pre-delivery	Post-delivery	Pre-delivery	Post-delivery
“Feels cool” (°C)	28.6	20.0	23.6	25.7
“Feels warm” (°C)	42.8	48.7	47.6	46.4
“Painfully cold” (°C)	14.5	20.6	9.7	17.9
“Painfully hot” (°C)	48.0	46.6	>50.0	>52.0
Mechanical detection threshold (Von Frey filament, g)	0.04	0.65	0.02	0.99

Two months later, four months after delivery, she reported troubling continuous buzzing and electric shocks in both legs, and a vice-like squeezing in the pelvis when she walked: symptoms that are consistent with neuropathic pain ^3^. These symptoms did not respond to the anti-neuropathic drug gabapentin, and persist six years after the delivery. Further treatment has focused on physiotherapy and conservative measures such as pacing and activity management.

## Discussion

Neuropathic pain arises as a direct consequence of a lesion or disease affecting the somatosensory system, and is characterized according to four criteria: pain distribution; the link between distribution and history; confirmatory tests of neurologic status demonstrating sensory signs confined to the territory of the lesioned nerve, and further confirmatory diagnostic tests to identify the lesion or disease entity underlying the neuropathic pain ^3^. The history, examination and investigations that we have described fulfill these criteria. We therefore believe that this patient has definite neuropathic pain, although it is manifested mostly by numbness, tingling, electric shocks and pressure, rather than stabbing or burning, but is nonetheless becoming increasingly debilitating.

It could be argued that the sensations described by our patient do not represent pain as understood by those without CIP. The International Association for the Study of Pain (IASP), however, defines pain as an unpleasant sensory and emotional experience associated with actual or potential tissue damage, or described in terms of such damage. The IASP also acknowledges that application of the word ‘pain’ is learnt in early life ( http://www.iasp-pain.org/Taxonomy?navItemNumber=576#Pain). There is psychometric and neuroimaging evidence that patients with CIP understand what the word ‘pain’ means, as they are able to empathize with behavioral and verbal expressions of pain in normal individuals ^4^.

Since her injury, our patient has started using a variety of means of describing pain with which most will be familiar. She reports that her right hip and pelvis still “hurt” a great deal, using descriptors such as “tight” and “aching”, and that she “suffers” if she walks too far or doesn’t wear an orthotic heel raise. At rest, these symptoms resolve, but she is left with “tingling”, “buzzing” and “electric shocks”. Furthermore she also describes headaches that respond to acetaminophen, “the sting of a graze”, “the sharpness of an exposed gum”, and “back aches”, “period pains” and “stomach cramps” that arose after pregnancy.

Our patient has also exhibited behavior consistent with a person in pain. She sought treatment in the local Pain Clinic (with DWW) for her symptoms, which suggests that these “buzzing” and “vice-like” descriptors had strong aversive-motivational qualities. We contend that these descriptions, in the context of the injury sustained and the resultant behaviors, are adequate to fulfill the IASP definition of pain, and have acknowledged and managed her report of pain as such.

A visual analog or numeric rating scale (NRS) is undoubtedly useful in normal individuals, and our patient rates her current pain intensity in the right hip and leg as between 0 and 4 on a 10-point NRS. However, the scale should not be required to validate the report of pain by this patient. Our patient currently scores 5 in the Douleur Neuropathique 4 (DN4) diagnostic neuropathic pain questionnaire, answering ‘yes’ to the presence of electric shocks, tingling and numbness, and having documented hypoesthesia to touch and pinprick in the affected area five years after the original injury ^5^. A score >4 provides 90% specificity for neuropathic pain in individuals with premorbid normal nociceptive physiology, but we judge that the diagnosis of neuropathic pain in our patient stands independently of the DN4 score, and question the value of neuropathic pain questionnaires in patients with CIP.

## Conclusion

The Nav1.7 channel plays a crucial role in pain transmission; however, this case shows that neuropathic pain can be initiated and maintained in its absence in humans, as well as in knockout mice ^6^, although we cannot rule out that Nav1.7 may mediate sharp or burning sensations. Our data provide a further rational basis for seeking specific molecular substrates for neuropathic pain, some of which could act as mechanistic targets for new therapies for patients with symptoms of neuropathic pain.
