# Supplementary material for: Case Report: Neuropathic pain in a patient with congenital insensitivity to pain
Source: F1000Res. 2015 Jun 19;3:135. Originally published 2014 Jun 26. [Version 2] doi: 10.12688/f1000research.2642.2 (PMC4670000; doi:10.12688/f1000research.2642.2)
Supplement: Peer review history

# Peer review: Referee response for version 2

**DOI:** 10.5256/f1000research.7158.r9123

**Published:** 2015-07-29

**Authors:** Juan D. Ramirez

**Affiliations:** 1 Nuffield Department of Clinical Neurosciences, University of Oxford, Oxford, UK

The authors have addressed the points thoroughly and added substantial evidence to support their claims. It is a well documented report on such a unique condition.

I have read this submission. I believe that I have an appropriate level of expertise to confirm that it is of an acceptable scientific standard.

# Peer review: Referee response for version 2

**DOI:** 10.5256/f1000research.7158.r9122

**Published:** 2015-06-22

**Authors:** Frank van Eijs

**Affiliations:** 1 Department of Anaesthesiology, St. Elisabeth Hospital, Tilburg, Netherlands

Congratulations with your work.

I have read this submission. I believe that I have an appropriate level of expertise to confirm that it is of an acceptable scientific standard.

# Peer review: Referee response for version 1

**DOI:** 10.5256/f1000research.2861.r6088

**Published:** 2014-09-17

**Authors:** Juan D. Ramirez

**Affiliations:** 1 Nuffield Department of Clinical Neurosciences, University of Oxford, Oxford, UK

This is an exciting report by Wheeler *et al.*, assessing the meaning of pain after a traumatic labour in a subject who has never experienced anything similar. This case is paramount for highlighting the importance of a detailed study of patients with inherited channelopathies.

Neuropathic pain has been established by IASP as pain arising as a consequence of damage to the somatosensory system. In this case the patient describes positive sensory phenomena in the form of buzzing and squeezing which is troubling and is accompanied by numbness which is often regarded as negative phenomena (positive and negative sensory abnormalities often co-exist in neuropathic pain states) so I would recommend changing the terms ‘sensory loss’ for both ‘sensory gain and loss’.

One issue is whether what the subject describes is more akin to paraesthesia rather than pain as understood by healthy subjects in whom NaV 1.7 is functional. The IASP definition of pain is actually fairly broad including the term ‘unpleasant sensory experience’. In the current case this sensory disturbance is an anatomically plausible distribution with evidence of a lesion to the somatosensory system.

The evidence comes from MRI that shows nerve root compression as a consequence of a haematoma. It would be helpful to have more sequences in order to demonstrate bilateral involvement and neurophysiology to define the extent of the nerve damage (sensory and motor).

The thermal and mechanical sensory testing is thorough and I would only recommend the authors to add the baseline temperature at which they started the assessment e.g. “32°C” added as part of the figure legend.

Finally, I would vouch for the use of the neuropathic pain symptom inventory for assessing in more detail the symptomatology of the subject ( Bouhassira *et al.,* 2004).

In general I believe this a relevant case that illustrates the need to carefully delineate new sensory symptoms in patients with congenital inability to experience pain and emphasises the distinct nature of neuropathic versus nociceptive pain states.

I have read this submission. I believe that I have an appropriate level of expertise to confirm that it is of an acceptable scientific standard, however I have significant reservations, as outlined above.

# Response

**Published:** 2015-06-12

**Authors:** Daniel W. Wheeler

**Affiliations:** Division of Anaesthesia, University of Cambridge, UK

- *This is an exciting report by Wheeler et al., assessing the meaning of pain after a traumatic labour in a subject who has never experienced anything similar. This case is paramount for highlighting the importance of a detailed study of patients with inherited channelopathies.*
  Thank you for your positive comments about our manuscript.
- *Neuropathic pain has been established by IASP as pain arising as a consequence of damage to the somatosensory system. In this case the patient describes positive sensory phenomena in the form of buzzing and squeezing which is troubling and is accompanied by numbness which is often regarded as negative phenomena (positive and negative sensory abnormalities often co-exist in neuropathic pain states) so I would recommend changing the terms ‘sensory loss’ for both ‘sensory gain and loss’.*
  We agree, and the text has been amended as suggested.
- *One issue is whether what the subject describes is more akin to paraesthesia rather than pain as understood by healthy subjects in whom NaV 1.7 is functional. The IASP definition of pain is actually fairly broad including the term ‘unpleasant sensory experience’. In the current case this sensory disturbance is an anatomically plausible distribution with evidence of a lesion to the somatosensory system.*
*The evidence comes from MRI that shows nerve root compression as a consequence of a haematoma. It would be helpful to have more sequences in order to demonstrate bilateral involvement and neurophysiology to define the extent of the nerve damage (sensory and motor).*
  The patient was seen in the clinical setting. Routine neurological examination revealed sensory loss to touch already suggesting neuropathy. EMG and ENG were not performed, as neither would have altered the clinical management in this case.
  Please see our response to Dr van Eijs for our comment on the MRI scan.
- *The thermal and mechanical sensory testing is thorough and I would only recommend the authors to add the baseline temperature at which they started the assessment e.g. “32°C” added as part of the figure legend.*
  The table caption has been amended to indicate that the baseline temperature was 32°C.
- *Finally, I would vouch for the use of the neuropathic pain symptom inventory for assessing in more detail the symptomatology of the subject ( Bouhassira et al., 2004).*
  Please see our response to Dr van Eijs regarding the value of pain questionnaires in patients with CIP.

# Peer review: Referee response for version 1

**DOI:** 10.5256/f1000research.2861.r5741

**Published:** 2014-09-11

**Authors:** Frank van Eijs

**Affiliations:** 1 Department of Anaesthesiology, St. Elisabeth Hospital, Tilburg, Netherlands

The presented aspects of the reported case are indeed interesting, however...

One cannot talk about pain if no pain is reported. Buzzing and a vice like squeezing of the pelvis is not the same as pain. The vice like squeezing of the pelvis may also have been due to the observed fractured pelvis. It is unclear if there indeed is subjective pain. If there is a report of pain there should also be an assessment of the visual analogue or numerical pain rating scale. Unfortunately this is not mentioned. Another clinical aspect is the missing of a DN4 score (douleur neuropathique 4 questionnaire). As I can see the score may not be more than 3 in which case the presence of neuropathic pain is unlikely.

The MRI scan of the L5-S1 interspace is not clear. It lacks a saggital view. The cross-sectional view should be more clearly and preferentially showing more than 1 slice. 

Suggestions:

1. Add the VAS or NRS of the pain intensity (probably 0?) Specify if pain scores are for the feet, legs or pelvis. If pain scores are not 0 then indeed there may possibly be neuropathic pain. In that case add the DN 4 score.
2. If there is no pain, symptoms should be mentioned as neuropathic or neuropathy instead of neuropathic pain. In that case also the title needs to be changed (e.g. neuropathy in a patient with congenital insensitivity to pain).
3. Add the saggital view of the lumbar MRI. Show more than 1 cross-sectional view of the MRI. Specify if views are T1, T2 or contrast dye enhanced.

I have read this submission. I believe that I have an appropriate level of expertise to confirm that it is of an acceptable scientific standard, however I have significant reservations, as outlined above.

# Response

**Published:** 2015-06-12

**Authors:** Daniel W. Wheeler

**Affiliations:** Division of Anaesthesia, University of Cambridge, UK

- *The presented aspects of the reported case are indeed interesting; however, one cannot talk about pain if no pain is reported. Buzzing and a vice like squeezing of the pelvis is not the same as pain. The vice like squeezing of the pelvis may also have been due to the observed fractured pelvis. It is unclear if there indeed is subjective pain. If there is a report of pain there should also be an assessment of the visual analogue or numerical pain rating scale. Unfortunately this is not mentioned. Another clinical aspect is the missing of a DN4 score (douleur neuropathique 4 questionnaire). As I can see the score may not be more than 3 in which case the presence of neuropathic pain is unlikely.*
*The MRI scan of the L5-S1 interspace is not clear. It lacks a sagittal view. The cross-sectional view should be more clearly and preferentially showing more than 1 slice. *
*Suggestions:*
*1. Add the VAS or NRS of the pain intensity (probably 0?) Specify if pain scores are for the feet, legs or pelvis. If pain scores are not 0 then indeed there may possibly be neuropathic pain. In that case add the DN 4 score.*
*2. If there is no pain, symptoms should be mentioned as neuropathic or neuropathy instead of neuropathic pain. In that case also the title needs to be changed (e.g. neuropathy in a patient with congenital insensitivity to pain)*
**Response**
  The International Association for the Study of Pain (IASP) defines pain as an unpleasant sensory and emotional experience associated with actual or potential tissue damage, or described in terms of such damage. The IASP also acknowledges that application of the word ‘pain’ is learnt in early life ( http://www.iasp-pain.org/Taxonomy?navItemNumber=576#Pain). It is unclear what patients have been diagnosed with congenital insensitivity to pain (CIP) understand by the word ‘pain’. However, there is psychometric and neuroimaging evidence they understand what the word ‘pain’ means, as they are clearly able to empathise with behavioural and verbal expressions of pain in normal individuals ( Danziger *et al.*, 2009).
  Our patient reports that her right hip and pelvis still “hurt” a great deal, using descriptors such as “tight” and “aching”, and that she “suffers” if she walks too far or doesn’t wear an orthotic heel raise. At rest, this “pain” resolves, but she is left with “tingling”, “buzzing” and “electric shocks”. **The patient in our report labelled her sensations as painful and we contend that description alone is adequate to fulfil the IASP definition of pain. **Furthermore she also describes headaches that respond to paracetamol, “the sting of a graze”, “the sharpness of an exposed gum”, and “back aches”, “period pains and stomach cramps” that arose after pregnancy.
  Our patient has also exhibited behavior consistent with a person in pain. She sought treatment in the local Pain Clinic (with DWW) for the sensations, which suggests that these “buzzing” and “vice-like” descriptors had strong aversive-motivational qualities. We believe that the simple verbal use of the word pain and behavioral response sufficed and have acknowledged and managed her report of pain as such. The visual analog or numeric rating scale (NRS) is undoubtedly useful in normal individuals, and our patient rates her current pain intensity in the right hip and leg as between 0 and 4 on a 10-point NRS. However, the scale should not be required to validate the report of pain by this patient.
  The DN4 score aims to increase the specificity of a diagnosis of neuropathic pain, with a score >4 providing 90% specificity for neuropathic pain. The sensitivity of the questionnaire is not well documented and the specificity of lower scores for neuropathic pain is unclear. The score in our patient was 5, but as the scale was validated **in individuals with premorbid normal nociceptive physiology**, we judge that the diagnosis of neuropathic pain stands independently of the DN4 score.
  We have added several additional paragraphs to the manuscript to address these points, which we hope you will find satisfactory.
- *Add the sagittal view of the lumbar MRI. Show more than 1 cross-sectional view of the MRI. Specify if views are T1, T2 or contrast dye enhanced.*
**Response**
  The MRI scans are T2-weighted and non-contrast enhanced. An additional cross-sectional view and a sagittal view are included below (included as Figure 1c and 1d in the revised manuscript). The arrows indicate the presence of hematoma material in the psoas and adjacent to the cauda equina.
